# Susceptibility of Chickens to Porcine Deltacoronavirus Infection

**DOI:** 10.3390/v11060573

**Published:** 2019-06-21

**Authors:** Qingqing Liang, Honglei Zhang, Bingxiao Li, Qingwen Ding, Yabin Wang, Wenming Gao, Donghui Guo, Zhanyong Wei, Hui Hu

**Affiliations:** 1 Department of Preventive Veterinary Medicine, College of Animal Science and Veterinary Medicine, Henan Agricultural University, Zhengzhou 450002, Henan, China; Liangqingqing180@126.com (Q.L.); hongleizhang2012@163.com (H.Z.); lbx0625@163.com (B.L.); 15083231067@139.com (Q.D.); Ybwang8686@126.com (Y.W.); 2 Key Laboratory for Animal-derived Food Safety of Henan Province, Zhengzhou 450002, Henan, China; 3 Henan Qixiang Biological Technology Co., Ltd, Jiaozuo 454000, Henan, China; 15803826941@126.com (W.G.); guodonghui0808@126.com (D.G.)

**Keywords:** porcine deltacoronavirus (PDCoV), chicken embryos, SPF chickens, cross-species transmission

## Abstract

Porcine deltacoronavirus (PDCoV) is a novel swine enteropathogenic coronavirus with worldwide distribution. PDCoV belongs to the *Deltacoronavirus* (DCoV) genus, which mainly includes avian coronaviruses (CoVs). PDCoV has the potential to infect human and chicken cells in vitro, and also has limited infectivity in calves. However, the origin of PDCoV in pigs, the host range, and cross-species infection of PDCoV still remain unclear. To determine whether PDCoV really has the ability to infect chickens in vivo, the three lines of chicken embryos and specific pathogen free (SPF) chickens were inoculated with PDCoV HNZK-02 strain to investigate PDCoV infection in the current study. Our results indicated that PDCoV can infect chicken embryos and could be continuously passaged on them. Furthermore, we observed that PDCoV-inoculated chickens showed mild diarrhea symptoms and low fecal viral RNA shedding. PDCoV RNA could also be detected in multiple organs (lung, kidney, jejunum, cecum, and rectum) and intestinal contents of PDCoV-inoculated chickens until 17 day post-inoculation by real-time quantitative PCR (qRT-PCR). A histology analysis indicated that PDCoV caused mild lesions in the lung, kidney, and intestinal tissues. These results prove the susceptibility of chickens to PDCoV infection, which might provide more insight about the cross-species transmission of PDCoV.

## 1. Introduction

*Coronaviruses* (CoVs) are enveloped, single-stranded, positive-sense RNA viruses that can infect and cause diseases in avian and mammal species, including humans [1,2]. CoVs contain the largest known RNA genomes and can be genetically divided into four genera; namely, *Alphacoronavirus*, *Betacoronavirus*, *Gammacoronavirus*, and *Deltacoronavirus* [3]. *Deltacoronavirus* (DCoV) genus was mainly discovered in a variety of avian species and pigs [4,5]. Porcine DCoV (PDCoV) was first detected in pigs during a molecular surveillance of CoVs in mammals and birds in Hong Kong in 2012 [5], while the first PDCoV outbreak in swine herds was reported in 2014 in the United States [6,7,8]. Thereafter, PDCoV was rapidly identified in many countries, including China, Canada, South Korea, Lao People’s Democratic Republic, Thailand, and Vietnam [9,10,11,12,13]. PDCoV can cause severe diarrhoea, vomiting, and dehydration in suckling and nursing piglets, and the clinical symptoms are indistinguishable from those caused by porcine epidemic diarrhoea virus (PEDV) and transmissible gastroenteritis virus (TGEV) [14,15]. PDCoV has caused serious economic losses for the pig industries. However, there are no effective reagents and vaccines to control PDCoV.

The host range of CoVs is expanding from wildlife to humans; generally, mammals are thought to be the host for *Alphacoronavirus* and *Betacoronavirus*, while avian is considered to be the host for *Gammacoronavirus* and DCoV [5]. Some CoVs can be transmitted to different animal species, and subsequently adapt to the new host, even to humans. Examples include the severe acute respiratory syndrome CoV (SARS-CoV) and middle east respiratory syndrome CoV (MERS-CoV), which were transmitted by civet cats and camels, respectively, to humans, and caused great harm to humans [16,17,18]. The novel swine acute diarrhoea syndrome coronavirus (SADS-CoV) was also verified to be a bat-related CoV that was 98.48% identical in genome sequence to the bat HKU2-CoV, which originated from the same genus of horseshoe bats as SARS-CoV [19]. However, little was known about the natural reservoir of these CoVs [2,20]. DCoVs have been mainly found in wild birds in early research, but recent findings suggested that DCoVs are also present in mammals [4,21,22,23]. With the detection of PDCoV in pig herds, it was confirmed that PDCoV had a close relation to birds’ DCoV, especially with sparrow CoV HKU17, which suggested there might exist bird-to-mammal transmission of PDCoV [5,24]. Meanwhile, DCoVs were detected previously in some small wild mammals, such as Asian leopard cats and Chinese ferret badgers. The S genes of the DCoVs isolated from these mammals were closely related to those of PDCoV identified in pigs, with the nucleotide similarity over 99.8% [5]. On the basis of these findings, we deduce that the potential interspecies transmission of PDCoV may exist between these wild small mammals and pigs, as well as between domestic pigs and birds/avian. 

To date, the origin of the novel PDCoV is still unknown. It was reported that PDCoV can infect 3- to 7-day-old gnotobiotic (Gn) calves with a high level of viral RNA titer in feces and specific IgG antibody in sera, but did not show any clinical symptoms and histological lesions [25]. Recently reports showed that PDCoV could infect human and chicken cells in vitro, indicating that PDCoV has the potential to infect chickens and humans to some extent [26]. Given the broad host ranges of PDCoV, we speculate that PDCoV may infect other mammalian and avian species. 

Whether the PDCoV really has the ability to infect chickens in vivo, and the exact mechanisms of the interspecies transmission and the pathogenicity of this novel virus, is largely unknown. Thus, in our study, the chicken embryos and specific pathogen free (SPF) chickens were used to evaluate the PDCoV infection. Our data indicated that PDCoV can infect three lines of chicken embryos, including White-feather Broiler, Hyline Layer, and SPF chicken embryos, and PDCoV can be passaged on these chicken embryos. Furthermore, we investigated the susceptibility of chickens to PDCoV infection. The PDCoV-inoculated chickens showed mild diarrhea symptoms; positive PDCoV RNA in feces and multiple organs; and mild histology lesions in the lung, kidney, and intestinal tissues. Our data suggested that chickens are susceptible to PDCoV infection, indicating that PDCoV may have the potential for cross-species transmission between pigs and chickens.

## 2. Materials and Methods

### 2.1. Cells and Virus

The LLC-porcine kidney (LLC-PK) cells were used to serially passage and propagate PDCoV in the current study. Cells were grown in minimum essential medium (MEM) (Gibco, Beijing, China) as described previously [27]. 

The virulent PDCoV HNZK-02 strain (GenBank accession number MH708123) was isolated from the intestinal contents of a piglet with diarrhea in Henan province by our laboratory in 2018, and this strain can cause disease in piglets with the symptom of severe diarrhea [28]. The passage 5 (P5) of this strain was used in the current study. The propagated PDCoV culture was harvested when the the cytopathic effect (CPE) was >85%, and confirmed negative for PEDV, TGEV, porcine rotaviruses (groups A, B, and C), porcine circovirus type 2 (PCV-2), and porcine reproductive and respiratory syndrome virus (PRRSV) by virus-specific PCRs. Virus titer was determined using TaqMan real-time quantitative RT-PCR (qRT-PCR) and by plaque assay, as described previously [27].

### 2.2. Experimental Animals

The SPF chicken embryos and SPF chickens were purchased from Jinan SIPAFAS poultry Co., Ltd in Jinan, China, and they met the national SPF chicken embryos standards requirement in production and inspection in the pharmacopoeia of the People’s Republic of China (2015). The White-feather Broiler and Hyline Layer chicken embryos were purchased from Henan Yue Poultry Farm (Henan, China). The chicken embryos were hatched in our lab. All the chickens were reared in animal isolators in Henan Qixiang Biological Technology Co., Ltd, and provided with sterilized feed and water. The experimental protocols and housing facility were approved by the Laboratory Animals Ethics Committee at Hennan Qixiang biological technology co.,LTD (approval ID: P2018001, approval date: April 20th, 2018).

### 2.3. Chicken Embryos’ Inoculation with PDCoV Strain HNZK-02

Fifteen embryos of each line (SPF, White-feather Broiler, and Hyline Layer chicken embryo) were used for PDCoV inoculation, and fifteen embryos were used as the negative control at each passage. Briefly, 11-day-old chicken embryos were inoculated via the allantoic cavity route with 8.62 log_10_ genomic equivalents (GE) of PDCoV and incubated at 37 °C. Embryo viability was examined using an egg candler. After three days of incubation, embryos were chilled for at least 6 h at 4 °C. The egg surface was cleaned with 75% ethanol, and then the eggshells above the air sac and the chorioallantoic membrane were carefully opened. The allantoic fluid was harvested, and the intestinal tissue of the chicken embryo was obtained by autopsy using the sterile scissors and forceps. The collected intestinal tissues were homogenated for the PDCoV viral RNA detection and defined as P0 generation. Next, the collected viral supernatants from the intestinal tissues were subjected to continuous passaging in the corresponding lines of chicken embryos. The experiment was repeated three times.

### 2.4. Chickens’ Inoculation with PDCoV Strain HNZK-02

Ninety four-day-old SPF chickens were randomly divided into two groups and housed in two separated isolators. Sixty chickens in group 1 were infected with PDCoV. The thirty chickens in group 2 were treated with MEM and served as uninfected controls. Prior to the viral challenge, the feces and blood for serum were collected for virus detection from three chickens of each group. Each chicken was intragastrically inoculated with 300 μL of PDCoV (10.5 log_10_ GE per chicken) or MEM.

The chickens were observed and evaluated daily for temperature changes and clinical signs until 17 day post-inoculation (dpi). Fecal samples were collected daily from each group for virus detection. Fecal scoring was recorded using a four-tiered system: 0 = normal feces, 1 = soft but formed feces, 2 = semi-fluid feces, and 3 = watery diarrhea, with scores of 2 or more considered diarrhea.

### 2.5. Sample and Tissue Collection

To evaluate viral shedding, fecal samples were collected each day from challenged and control chickens for PDCoV detection until 17 dpi. For each group, three chickens were selected for necropsy at 3, 5, 7, 10, 14, and 17 dpi, respectively. The chickens’ intestinal contents and serum were collected for virus detection. All the collected fecal samples and intestinal contents were diluted five-fold with MEM and centrifuged at 1847g at 4 °C for 20 min. The supernatants were collected for viral RNA extraction.

Fresh and formalin-fixed samples, including the heart, liver, spleen, lung, kidney, duodenum, jejunum, ileum, cecum, rectum, and pancreas, were collected during the chicken necropsy. The fresh samples were used for viral distribution assay and the formalin-fixed tissues served as the pathologic examination.

### 2.6. Viral RNA Extraction and RT-PCR, qRT-PCR 

Tissue tropism of PDCoV in experimentally infected chickens was tested by conventional RT-PCR, and dynamic distribution of PDCoV in feces and various tissues was determined by qRT-PCR. Briefly, total RNAs were extracted from the intestinal content suspensions and tissues samples using Trizol reagent (Invitrogen, Carlsbad, CA, USA), according to the manufacturer’s instructions. Reverse transcription (RT) was conducted using the Vazyme Reverse Transcription Kit (Nanjing, China). The primers targeting the PDCoV S gene (PDCoV-S609-F: 5’-CAACCGTCTTGAGGAAGTAGAG-3’ and PDCoV-S609-R: 5’-TCAACGGTGAGGTTGAGAATAG-3’) were designed based on the sequence of the USA/Iowa136/2015 strain (GenBank accession no. KX022602), and the amplified fragment was 609 bp. The cDNA was amplified using Taq DNA Polymerase (TaKaRa, Dalian, China). The PCR products were sequenced twice using the forward and reverse PCR primers. Viral RNA titers was performed by qRT-PCR as reported previously [27]. qRT-PCR was conducted using the Premix Ex Taq^TM^ (Probe qPCR) kit (TaKaRa, Dalian, China) on a real-time thermocycler (CFX96^TM^ Optics Module, BIO-RAD), and the results were analyzed using the system software. The detection limit of the qRT-PCR was 10 GEs/reaction, which was corresponded to 4.6 log10 GE/mL of PDCoV in fecal and 3.6 log10 GE/mL in serum samples, respectively.

For each conventional RT-PCR and qRT-PCR assay, 10-fold dilutions of standard plasmid were generated as the positive control, and negative control samples and double-distilled water (ddH_2_O) were also included.

### 2.7. Histology

Chicken intestinal tissues and other major organs (lung, liver, heart, kidney, and spleen) were examined grossly and histologically, and routinely fixed in 10% (*vol/vol*) phosphate-buffered formalin for 48 h at room temperature [29]. They were then embedded, sectioned, and stained with hematoxylin and eosin (H&E). Slides were examined for light microscopy examination by conventional light microscopy.

### 2.8. Chicken-PDCoV S Gene Sequencing and Phylogenetic Analyses

The complete S gene of PDCoV in the SPF chicken-passaged samples was amplified, cloned, and sequenced. The S genes were amplified using primers PDCoV-S-F4 (5’-CAGGACGCCTTCTTGTGA -3’) and PDCoV-S-R4 (5’-GGGTTCGGCTTGGAGTAG -3’). The primers were designed based on the sequence of the PDCoV strain HKU15-155, and the expected size of the amplicon was 3692 bp. Total RNA was extracted from the tissue samples and converted to cDNA by oligo (dT)-priming strategy (Nanjing, China), as described above. The S gene was amplified using PrimeSTAR GXL DNA Polymerase (Takara). The PCR mixture (50 μL) contained cDNA 2 μL, 5 × PCR buffer 10 μL, dNTP (2.5mM each) 4 μL, PCR enzyme GXL 1 μL, and forward and reverse primers (50μM stock) 1 μL each. The PCR program was as follows: 98 °C for 5 min, 40 cycles of 98 °C for 20 s, 55 °C for 15 s, 68 °C for 4 min, and 68 °C for 10 min. The amplified fragment was sequenced and assembled using DNAStar 7.0 green (DNAstar, Madison, WI, USA). The sequence data were subjected to phylogenetic analysis, according to our previously reported methods [27]. 

The PDCoV HNZK-02 strain passaged in chicken was named as PDCoV CC-HNZK-02 strain. The complete S gene sequence of PDCoV CC-HNZK-02 strain was deposited in GenBank under accession number MK248485.

## 3. Results 

### 3.1. PDCoV Can Infect Chicken Embryos

Three different lines of chicken embryos were infected with PDCoV HNZK-02 by allantoic cavity inoculation. The intestinal tissues from the same viral-inoculated embryos were collected and homogenated, and subjected to continuous passaging in the corresponding embryos. PDCoV was passaged for six rounds in broiler embryos and for two rounds in SPF embryos, respectively. The PDCoV RNAs were identified in some passages by RT-PCR and qRT-PCR (Table 1). Our results showed that the PDCoV RNAs could be detected in these three kinds of embryos at each passage, but it did not induce visible pathological changes and death in embryos. The peak viral RNA titers of the PDCoV HNZK-02 propagated in the three kinds of embryos were similar (around 6.35~6.70 log_10_ GE/mL) (Table 1). However, the growth kinetics of the PDCoV in the three kinds of embryos were quite different. The PDCoV RNA titer reached a peak at the first passage in the SPF and layer chicken embryos, and then started to decrease thereafter. The viral RNA titer peaked after one passage in the broiler embryos and then decreased significantly, but it increased at the later passage.

### 3.2. Clinical Signs of the PDCoV-Challenged Chickens

**Figure 1:**
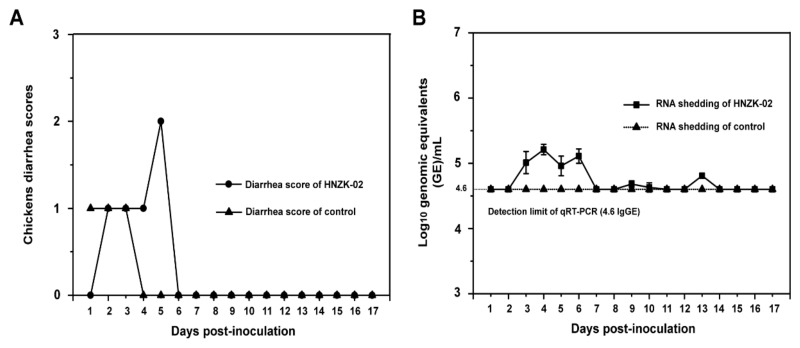
Diarrhea scores (A) and fecal viral RNA shedding (B) in specific pathogen free (SPF) chickens inoculated with Porcine deltacoronavirus (PDCoV) HNZK-02. Four-day-old SPF chickens were inoculated with PDCoV HNZK-02 (10.5 log_10_ genome equivalent (GE) of PDCoV per chicken, *n* = 60). (**A**) Clinical signs were monitored daily, and diarrhea was scored for each chicken as follows: 0 = normal feces, 1 = soft but formed feces, 2 = semi-fluid feces, and 3 = watery diarrhea, with scores of 2 or more considered diarrhea. (**B**) The fecal RNA shedding titers were determined by real-time quantitative RT-PCR (qRT-PCR). The detection limit of the qRT-PCR was 4.6 log_10_ GE/mL of PDCoV in fecal samples. Three independent experiments were performed and there were five biological replicates at each group. The data were shown as mean values of the quintuplicate from a representative experiment with standard deviation error bars.

To evaluate whether PDCoV can infect and cause clinical symptom in chickens, the four-day-old SPF chickens were inoculated with PDCoV HNZK-02 and observed daily for 17 days. The group inoculated with PDCoV HNZK-02 showed only mild diarrhea at 5 dpi, indicating that the diarrhea symptoms caused by PDCoV were mild in chickens (Figure 1A). The negative control chickens exhibited no clinical signs throughout the course of the experiments. The body temperatures of all chickens were also recorded every day, and the data showed that there were no significant differences between the PDCoV-inoculated group and the control group. 

### 3.3. Viral Shedding and Distribution in PDCoV-Inoculated SPF Chickens

The fecal viral shedding was determined using qRT-PCR. PDCoV-specific viral RNA was first detected in fecal samples of the PDCoV HNZK-02-inoculated SPF chickens at 3 dpi, and fecal viral RNA peaked on 4 dpi, but it is still very low (around 5.5 log_10_ GE/mL), and negative at 7 dpi (Figure 1B). The negative control chickens did not shed detectable PDCoV viral RNA in feces throughout the experiment.

**Figure 2:**
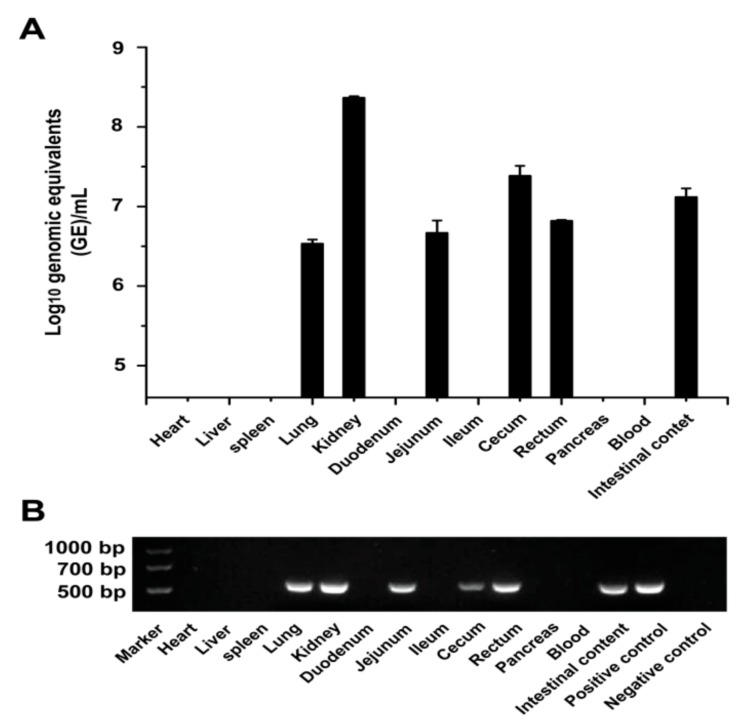
Virus distribution in PDCoV-inoculated chickens. (**A**) Virus distribution in PDCoV-inoculated SPF chickens at 5 dpi. Three independent experiments were performed and there were five biological replicates in each group. The data are shown as the mean values of the quintuplicate from a representative experiment with standard deviation error bars. (**B**) RT-PCR detection of the virus distribution in PDCoV-inoculated SPF chickens at 5 dpi.

To determine the PDCoV distribution in chicken tissues, the viral RNA was first tested from the collected tissue samples at 5 dpi by RT-PCR and qRT-PCR (Figure 2A,B). PDCoV viral RNAs were detected in the lung, kidney, jejunum, cecum, and rectum of the PDCoV-inoculated chickens (Figure 2A). The viral RNA level was relatively high in the kidney (8.5 log_10_ GE/mL) and cecum (7.5 log_10_ GE/mL). Further, no PDCoV RNA was detected in the heart, liver, spleen, pancreas, duodenum, and ileum. The relatively high level of PDCoV RNA was also detected in the intestinal contents (7.2 log_10_ GE/mL). The PDCoV HNZK-02 inoculated chickens were negative for PDCoV RNA in the sera. 

**Figure 3:**
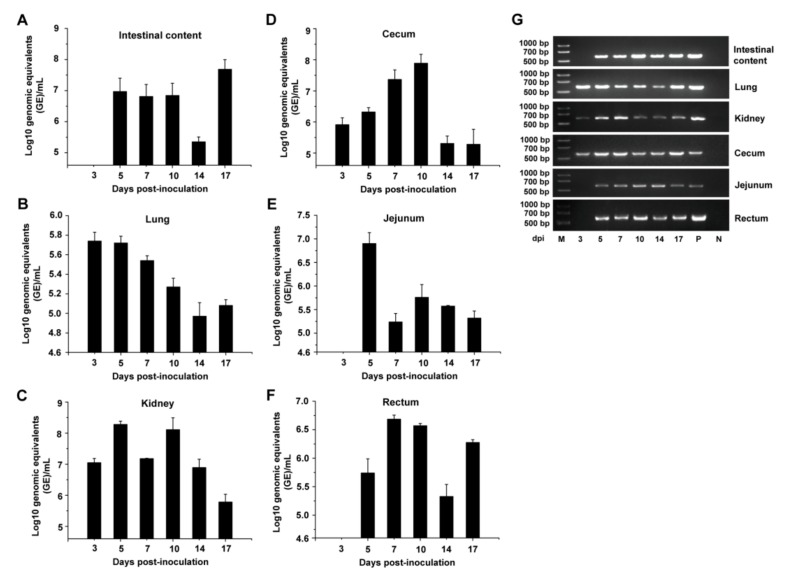
Viral shedding in tissues of the PDCoV-inoculated chickens at the indicated testing times. Viral RNA shedding in the intestinal contents (A), lung (B), kidney (C), cecum (D), jejunum (E), and rectum (F) at 3, 5, 7, 10, 14, and 17 dpi (*n* = 5). Three independent experiments were performed and there were five biological replicates in each group. The data are shown as mean values of the quintuplicate from a representative experiment with standard deviation error bars. (G) RT-PCR detection of the viral shedding in the intestinal contents, lung, kidney, cecum, jejunum, and rectum at the indicated times.

Dynamic distribution of PDCoV in intestinal contents and different tissues was further determined by qRT-PCR (Figure 3). Relatively high levels of PDCoV-specific viral RNA were detected until 17 dpi in intestinal content samples (Figure 3A). The viral RNA could be detected at 3 dpi (5.7 log_10_ GE/mL) in lungs, and the viral titer peaked at 3 to 5 dpi, and then decreased gradually thereafter, but still showed viral RNA positive at 17 dpi (Figure 3B). High levels of PDCoV RNA titer were detected almost throughout the experiment in the kidney (around 7.0~8.5 log_10_ GE/mL) and cecum (around 6.0~8.0 log_10_ GE/mL) (Figure 3C,D). PDCoV RNA titer reached a peak at 5 and 7 dpi in the jejunum and rectum, respectively, and then decreased thereafter. However, the viral RNA was not detected in the jejunum and rectum at 3 dpi (Figure 3E,F). As shown in Figure 3G, the expected 609 bp RT-PCR products were also detected in all these tissue samples and intestinal content samples at the indicated time.

### 3.4. Gross and Histologic Lesions in SPF Chickens Inoculated with the PDCoV HNZK-02 Strain

Macroscopic examination showed that PDCoV-inoculated SPF chickens had mild lesions characterized by thin cecum intestinal walls and accumulation of some yellow fluid in the intestinal lumen of the cecum. Other organs and tissues appeared normal. 

**Figure 4:**
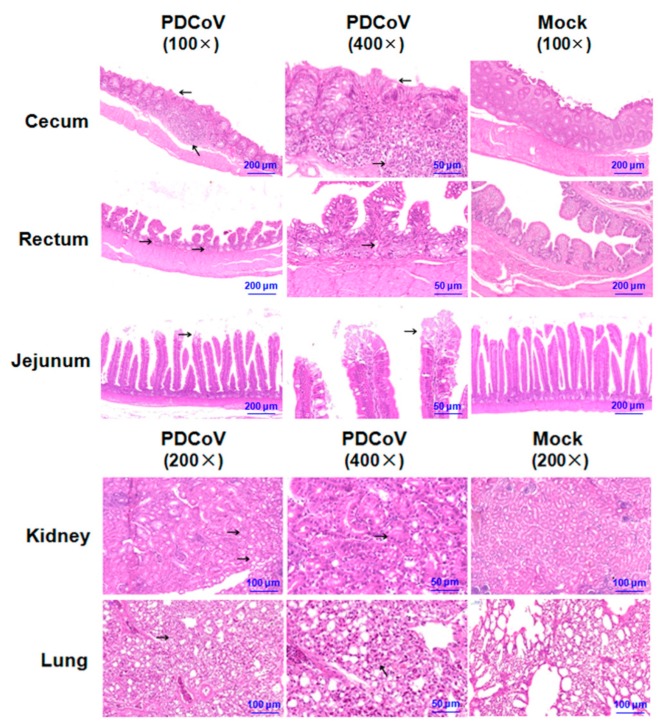
Histology of tissue sections from chickens inoculated with PDCoV HNZK-02. The tissues (cecum, rectum, jejunum, kidney, and lung) from the SPF chickens inoculated with PDCoV HNZK-02 and the control group were collected at 5 dpi and then stained via the hematoxylin and eosin method (H&E). The arrows indicate the typical histological lesions in the detected tissues. Scale bars are shown in each picture.

The tissues of the rectum, cecum, jejunum, lung, and kidney were collected at 5 dpi for histopathological analysis. As shown in Figure 4, these tissues showed obvious patterns of pathology in PDCoV-inoculated SPF chickens when compared with the mock-infected chickens. Histological lesions included necrosis of crypt cells in the rectum and mild intestinal villi shedding in the jejunum. The typical histological lesions in cecum were characterized by intestinal crypt cells with diffuse necrosis, lymphoid follicle expansion, and a large amount of lymphocyte infiltration. The obvious lesion in the kidney was characterized by degeneration and necrosis of renal tubular. In addition, the alveolar septum became thick and a small amount of lymphocytes was scattered in the alveolar space in the lung.

### 3.5. Characterization of the S Gene of PDCoV HNZK-02 after Inoculation in SPF Chickens

**Figure 5:**
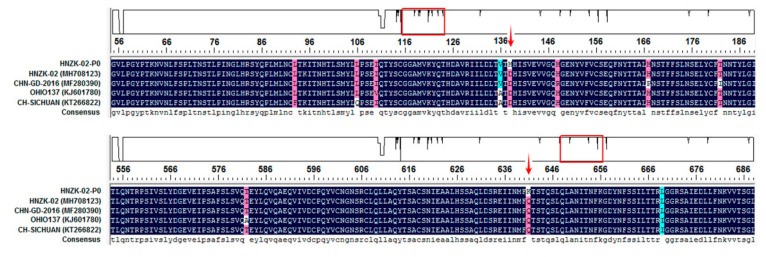
Amino acid locations of the PDCoV S protein mutations that developed upon virus passage in chickens. Alignment of S protein sequences in different PDCoV strains using the DNAStar 7.0 green (DNAstar, Madison, WI, USA). Reference S protein sequences obtained from GenBank are indicated by strain names and GenBank accession numbers. The two mutation amino acids from S protein in the chickens are indicated with red arrows. The highlighting box indicate the position of amino acids mutation in the S gene of the PDCoV CC-HNZK-02 strain compared with the reference strains.

To examine if genetic changes occurred in the S gene of PDCoV during passage in chickens, the complete S gene of PDCoV CC-HNZK-02 strain (PDCoV in the challenged chickens) was amplified and sequenced. The sequenced S gene was 3480 nucleotides long, encoding a protein of 1160 amino acids (aa). Compared with the cell-cultured PDCoV HNZK-02 strain, the PDCoV CC-HNZK-02 had 2 aa mutations in S gene: Asp changed to His at position 138, and Gln changed to Lys at position 641 (the aa positions were numbered according to the S gene of PDCoV HNZK-02 sequence) (Figure 5). Meantime, these two aa mutations also existed in some reference PDCoV strains, including CHN-GD-2016 strain (MF280390), OHIO137 strain (KJ601780), and CH-SICHUAN strain (KT266822) (Figure 5). Compared with these three reference PDCoV strains, there also existed other aa mutations (at positions 93, 106, 110, 136, 148, 167, 182, 582, and 669) in the S gene of the PDCoV CC-HNZK-02 strain.

**Figure 6:**
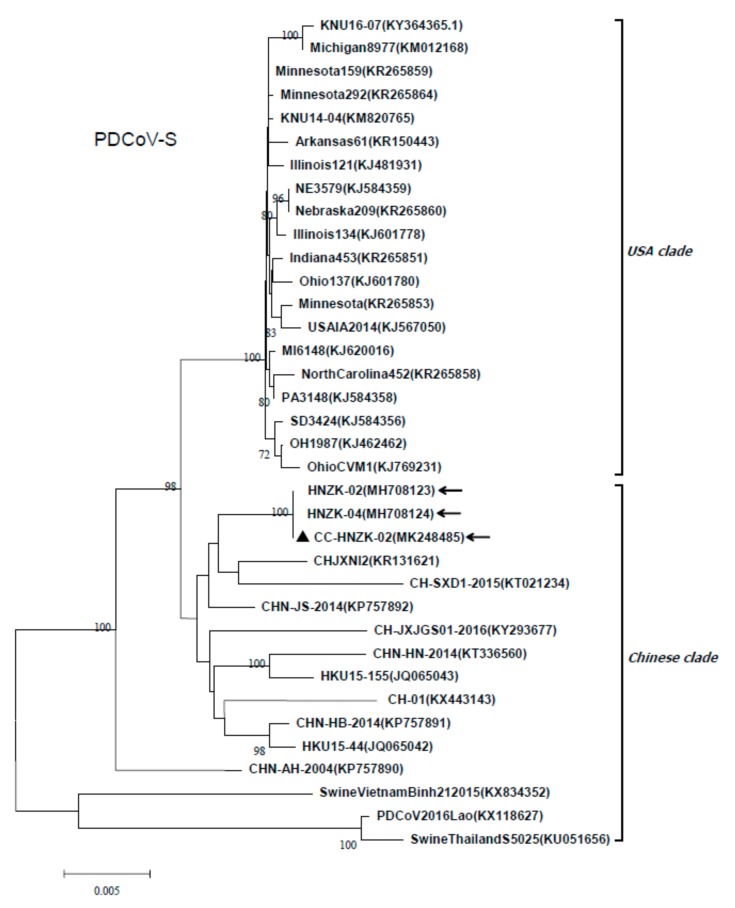
Phylogenetic analysis based on the S genes from different PDCoV strains. The phylogenetic tree was constructed from the aligned nucleotide sequences using the neighbour-joining method with MEGA 6.06 software. Bootstrap values were calculated with 1000 replicates. Reference sequences obtained from GenBank are indicated by strain names and GenBank accession numbers. The S gene of the PDCoV strain CC-HNZK-02 from chickens is indicated with black triangles. The arrows indicate the PDCoV strains that identified by our laboratory. Scale bars indicate nucleotide substitutions per site.

Phylogenetic analysis was carried out based on the S gene of the PDCoV CC-HNZK-02 and other PDCoVs obtained from GenBank. The result showed that the PDCoV strain CC-HNZK-02 from chickens was closely related with the PDCoV strain HNZK-02, and also closely related with most of the Chinese strains, including the HNZK-04 strain identified by our laboratory (Figure 6). 

## 4. Discussion

The cross-species transmission of viruses is responsible for most emerging infectious diseases, and can seriously affect the safety and health of human and animals. There exhibits an obvious tendency for CoVs to engage in cross-species transmission, like the SARS-CoV and MERS-CoV [26,30]. PDCoV is a novel swine intestine CoV and may have a broad host range [25]. However, it is still unclear whether PDCoV can infect avian species, including chickens and other poultry. In this study, we first inoculated the PDCoV HNZK-02 strain into three kinds of chicken embryos, and then the susceptibility of SPF chickens to PDCoV infection was investigated. Our study demonstrated that PDCoV can infect chicken embryos, and could be passaged on them. Moreover, SPF chickens intragastrically inoculated with the PDCoV strain HNZK-02 to develop a mild infection with persistent PDCoV RNA shedding in tissues and intestinal contents, and also showed mild intestinal lesions and clinical disease. Our results indicated that chickens are susceptible to PDCoV infection, but do not present with clinical signs, which might provide more insight about the cross-species transmission of PDCoV.

In our preliminary experiment, we found that PDCoV can grow in the DF-1 cell in vitro, and the PDCoV viral RNA could be detected at each passage over five rounds, but it did not induce a visible cytopathic effect (CPE). Recently, a study also showed that PDCoV can infect avian origin cells, including Leghorn male hepatoma (LMH) and DF-1 cell lines [26]. Thus, in the current study, we first tested if PDCoV could grow in chicken embryos. Our data showed that PDCoV could consecutively propagate in these three lines of chicken embryos, and the viral RNAs were detected even after six rounds of passage in the broiler embryos. Meanwhile, the relatively low PDCoV RNA titer also could be detected at some passages of another two kinds of chicken embryos, suggesting that there existed a lower replication of PDCoV in the chicken embryos, possibly because of its incomplete adaptation. 

To further evaluate whether PDCoV can infect and propagate in chickens, the SPF chickens were inoculated with the PDCoV HNZK-02 strain. These SPF chickens showed mild diarrhea symptoms and shed PDCoV RNAs in intestinal contents and tissues until 17 dpi, indicating that PDCoV could persistently infect SPF chickens. Furthermore, PDCoV-specific viral RNA was also detected in fecal samples until 6 dpi. Previous reports showed that PDCoV can infect gnotobiotic and conventional piglets, and caused severe atrophic enteritis characterized by severe watery diarrhea and vomiting [22]. However, PDCoV-infected pigs appeared to shed less PDCoV RNA titer in the feces when compared with the inoculated viral titer [21,22], implying a potentially lower replication rate of PDCoV in the gastrointestinal tract of pigs. In addition, a comparable low viral RNA titer was detected from fecal samples of gnotobiotic calves challenged with PDCoV [25], which also suggested that there was a lower replication of PDCoV in the intestine of calves. These findings raise the possibility that PDCoV is susceptible to piglets, calves, and chickens, but it may not yet be completely adapted to these animals. Studies are needed to investigate if the mortality of PDCoV infection in chickens could be increased by serially passaging PDCoV in chickens. 

Several reports showed that PDCoV RNA could be detected in low to moderate quantities in multiple organs (intestine, blood, lung, liver, and kidney) in PDCoV-infected piglets by qRT-PCR, indicating multi-systemic viral distribution occurred during PDCoV infection [7,21,31]. In this study, our results indicated that PDCoV viral RNA was mainly detected in chicken lungs; kidneys; and multiple intestinal tissues, including cecum, jejunum, and rectum, implying that there exhibits an extensive tissue tropism during PDCoV infection in chickens. Collectively, PDCoV could infect the lung tissue in pigs and chickens, suggesting that outside of the gastrointestinal tract, the respiratory tract may also be a target route for PDCoV infection. Further studies are still needed to investigate the exact transmission route of PDCoV in chickens.

Our previous report has verified that there existed the mutation of aa in PDCoV S genes during PDCoV passage in ST and LLC-PK cell cultures [27]. In the current study, PDCoV strain CC-HNZK-02 from the inoculated SPF chickens had 2 aa mutations in S gene when compared with the challenged HNZK-02 strain. The CoVs S protein is responsible for receptor binding and membrane fusion, and its receptor specificity and binding ability determine the cross-species transmission within the novel host [26]. It is still unknown whether passage PDCoV in chickens could increase its pathogenicity, and it needs to be studied. Further study of spike-receptor interactions may provide new insight into the cross-species transmission event of PDCoV.

**Figure 7:**
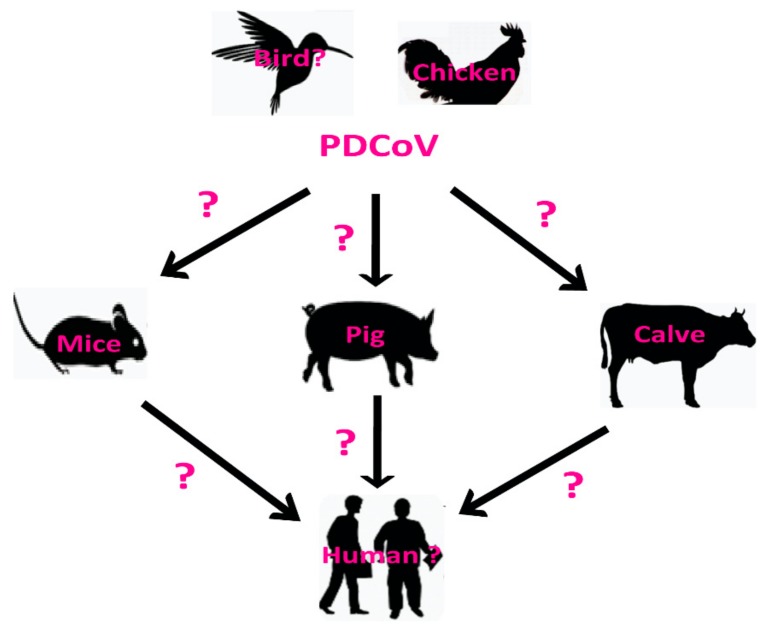
Schematic of the potential host range of Porcine deltacoronavirus (PDCoV) and suspected routes of inter-species transmission. The question marks in different species indicate potential, but unidentified hosts of PDCoV, including human and birds. The question marks on the arrows indicate suspected, but unidentified routes of PDCoV cross-species transmission.

PDCoV has become a common swine enteric pathogen with worldwide distribution and may have a potential broad host range, including humans and birds (Figure 7). To date, all the members of the DCoVs genus except PDCoV have been detected in birds. In our study, we found that the PDCoV can infect chickens, indicating that PDCoV may cross the species barrier from avian/birds to some mammals (Figure 7). However, our study can not demonstrate if PDCoV has the ability for direct interspecies transmission between pigs and chickens at present. The aminopeptidase N (APN) is a protein receptor, and is mainly targeted by a number of alphacoronaviruses, including TGEV and Human Coronavirus 229E (HCoV-229E) [26]. Recent reports also showed that PDCoV can efficiently infect human origin cells via interacting with the human APN receptor [26], suggesting that there are potential possibilities for PDCoV to cross the species barriers to infect humans (Figure 7). To define the natural host and/or reservoir of PDCoV, further molecular surveillance study and experiments on artificial infection of different animals with PDCoV are needed to investigate the interspecies transmission of PDCoV.

In conclusion, we have demonstrated that chicken embryos and SPF chickens are susceptible to PDCoV infection. PDCoV-inoculated chickens showed low fecal viral RNA shedding, extensive tissue tropism with moderate viral RNA titer, and mild lesions on intestinal tissues. However, there were no obvious clinical symptom in PDCoV-inoculated chickens. These results provide new insight into a novel host range and the cross-species transmission of PDCoV.

## Figures and Tables

**Table 1:** Detection of Porcine deltacoronavirus (PDCoV) propagation and passage in chicken embryos.

Embryo group	Inoculum Titer, log_10_ GE/Embryo	Viral RNA Shedding, log_10_ GE/mL *, by Passage							
		0	1	2	3	4	5	6	7
**Broiler Embryo**	8.62	5.68 ± 0.16	6.35 ± 0.06	5.52 ± 0.06	6.02 ± 0.07	5.99 ± 0.03	5.7 ± 0.06	6.2 ± 0.07 ^#^	ND *
**Layer chicken Embryo**	8.62	ND	ND	ND	ND	ND	6.61 ± 0.02	6.34 ± 0.09	5.69 ± 0.01
**SPF Embryo**	8.62	6.7 ± 0.11	6.07 ± 0.02	5.97 ± 0.15	ND	ND	ND	ND	ND
**Control**	-	<4.6	<4.6	<4.6	<4.6	<4.6	<4.6	<4.6	ND

* GE, genome equivalents; ND, not detected; SPF, specific pathogen free. ^#^ Detected using real-time quantitative RT-PCR (qRT-PCR) (*n* = 5). Detection limit of the qRT-PCR for intestinal samples were <4.6 log_10_ GE/mL.
